# Signaling Dynamics of TSHR-Specific CAR-T Cells Revealed by FRET-Based Biosensors

**DOI:** 10.3389/fcell.2022.845319

**Published:** 2022-02-17

**Authors:** Jing Zhou, Jiangqing Chen, Yanjie Huang, Xiaofei Gao, Chun Zhou, Xianhui Meng, Jie Sun

**Affiliations:** ^1^ Department of Breast and Thyroid Surgery, Union Hospital, Tongji Medical College, Huazhong University of Science and Technology, Wuhan, China; ^2^ Department of Breast and Thyroid Surgery, People’s Hospital of Dongxihu District Wuhan City and Union Dongxihu Hospital, Huazhong University of Science and Technology, Wuhan, China; ^3^ Liangzhu Laboratory, Zhejiang University Medical Center, Hangzhou, China; ^4^ Department of Cell Biology, Bone Marrow Transplantation Center of the First Affiliated Hospital, Zhejiang University School of Medicine, Hangzhou, China; ^5^ Institute of Hematology, Zhejiang University and Zhejiang Engineering Laboratory for Stem Cell and Immunotherapy, Hangzhou, China; ^6^ Key Laboratory of Structural Biology of Zhejiang Province, School of Life Sciences, Westlake University, Hangzhou, China; ^7^ School of Public Health, Sir Run Run Shaw Hospital, Zhejiang University School of Medicine, Hangzhou, China

**Keywords:** FRET, ERK, ZAP70, CAR-T, TSHR, thyroid cancer

## Abstract

Although most patients with thyroid cancers have good prognosis and long-term survival, some patients are refractory to traditional therapeutic approaches and face a high risk of mortality. CAR-T therapy provides an attractive strategy to treat these patients. Considering the limited expression in thyroid tissues, thyroid-stimulating hormone receptor (TSHR) has been considered as a promising candidate as CAR-T target. However, it is still a challenge to find the optimal CAR design for the treatment of thyroid cancers. Dynamic signaling cascade is initiated by CAR molecules during CAR-T cell activation. The development of FRET-based biosensors enables us to detect the signaling dynamics of key kinases during CAR-T cell activation with high spatiotemporal resolution. Here using the ZAP70 and ERK biosensors, we visualized the dynamics of ZAP70 and ERK activities in TSHR-specific CAR-T cells upon antigen stimulation. We first constructed several TSHR-targeting CARs for the treatment of advanced thyroid cancers. The TSHR CAR-T cells with CD28 or 4-1BB co-stimulatory signaling domains exhibited potent cytotoxicity *in vitro*. By FRET imaging, we observed rapid increase of ZAP70 and ERK activities in TSHR CAR-T cells upon target cell binding. Even though CD28-based CAR-T cells had similar ZAP70 activation dynamics as 4-1BB-based CAR-T cells, they displayed slightly enhanced ERK activation, which may contribute to their faster anti-tumor kinetics *in vivo*. These results demonstrated the efficacy of TSHR CAR-T cells to treat advanced thyroid cancers. Our study indicated the potential of applying FRET biosensors to optimize the design of CAR for effective CAR-T therapy.

## Introduction

Thyroid cancer is the most common endocrine cancer and accounts for 3% of the new cancer cases worldwide in 2020 (Sung et al., 2021). Majority of thyroid cancers are differentiated thyroid cancers (DTC, 94%), which mainly include papillary thyroid cancer (PTC, 80% of all cases), and follicular thyroid cancer (FTC, 15% of all cases). Although most patients with DTCs have excellent prognosis after radical surgery and radioactive iodine (RAI) treatment, a small fraction of them (about 20%) may develop into advanced stage DTCs after tumor recurrence and metastasis, which may require reoperation. Large amount of fibrous and scar tissue in the surgical field will bring difficulties to the reoperation, which increases the incidence of permanent nerve injury and hypoparathyroidism, especially for patients who needs central lymph nodes dissection and/or cervical lymph nodes dissection again. It is reported that the incidence of permanent hypocalcemia and permanent vocal cord paralysis after re-dissection of cervical lymph nodes can be as high as 4.9 and 17.8% respectively, which may seriously affect the quality of patients’ life. For such patients, exploring a new non-invasive treatment method is of great value. In addition, 5–10% of DTC cases with recurrence and metastasis are resistant to traditional therapy and suffer a high risk of mortality. It is reported that patients with refractory DTCs have less than 50% of 5-years survival rate (Laha et al., 2020). For these patients, it is urgent to explore new treatment methods.

Chimeric antigen receptor T (CAR-T) therapy represents a novel therapeutic approach and has achieved significant progress in treating hematological malignancies. The canonical CAR structure includes an scFv domain responsible for antigen recognition, a hinge and transmembrane domain, and a combined co-stimulatory and activation domain that initiates T cell activation. CAR molecules can reprogram T cell to recognize and eliminate tumor cells expressing specific antigens (van der Stegen et al., 2015). Although many challenges remain, experimental and clinical studies have shown some positive outcomes of CAR-T therapy for solid tumors (Newick et al., 2017). For advanced thyroid cancers, recent studies demonstrated that CAR-T therapy has potential therapeutic efficacy in treating anaplastic thyroid cancer (ATC) and medullary thyroid cancer (MTC) in preclinical models (Min et al., 2017; Bhoj et al., 2021).

Successful application of CAR-T therapy requires specific recognition of tumor cells. However, most solid tumors, including thyroid cancer, lack tumor-specific antigens that could be used to precisely distinguish tumor and normal tissue cells. Some tumor-associated antigens (TAA) have been used as CAR-T cell targets, while the risk of tumor relapse or severe on-target/off-tumor toxicity has to be evaluated carefully. As an alternative, lineage-specific antigens may be considered as targets of CAR-T cells in solid tumors as long as complete tissue eradiation is tolerant (Newick et al., 2017; Kosti et al., 2018). The thyroid-stimulating hormone receptor (TSHR) is a surface glycoprotein receptor whose expression is highly restricted to thyroid tissues. Evidence has shown continued high expression of TSHR in the majority of DTCs, including 90.8% in PTC, 89.2% in FTC, 78.2% in the cervical lymph node metastases, and 86.7% in the RAI-resistant metastases. These features suggest the potential of TSHR as CAR-T therapy targets for the treatment of advanced stage DTCs (Davies et al., 2002; Rowe et al., 2017).

Currently, the second-generation CARs that incorporate CD28 or 4-1BB signaling domains are the most prevalent CAR designs. Both CD28 and 4-1BB-based CAR-T cells can achieve complete tumor eradiation in clinical studies of refractory B cell malignancies. However, the different co-stimulatory domains have endowed CAR-T cells with distinct anti-tumor kinetics. CD28-based CAR-T cells often show rapid tumor eradiation with less persistence. In contrast, 4-1BB-based CAR-T cells often have less cytotoxic effect while persist longer *in vivo* (Feucht and Sadelain, 2020). Therefore, in the context of different solid tumors, the anti-tumor functions of CD28 and 4-1BB-based CARs may vary.

Nowadays, the development of genetically-encoded, FRET-based biosensors makes it possible to investigate kinetics of key signaling molecules in live cells with high spatiotemporal resolution. Recent studies have demonstrated that FRET-based biosensors can be used to monitor dynamics of some key molecules responsible for T cell activation (Xiang et al., 2011; Li et al., 2016; Wan et al., 2019). Particularly, CAR structures with modified CD3ζ domains were shown to induce different Zeta-chain-associated protein kinase 70 (ZAP70) activation kinetics in CAR-T cells upon antigen stimulation (Liu et al., 2021).

In this study, we designed CAR-T cells targeting TSHR and evaluated their efficacy for the treatment of metastatic thyroid cancers both *in vitro* and *in vivo*. Using FRET-based biosensors, we further quantified and compared the dynamics of ZAP70 and Extracellular signal-regulated kinase (ERK) signals in CD28 and 4-1BB-based CAR-T cells upon antigen stimulation. As a member of the Syk family of thrysine kinases, ZAP70 plays a crucial role in regulating T cells activation. After antigen engagement, ZAP70 is recruited to the phosphorylated CD3ζ subunits, where it is activated by LCK kinase and facilitates downstream signal amplification (Wang et al., 2010). Previous studies have suggested ZAP70 signal as a rate-limiting step in CAR-T cells activation. Insufficient activation of ZAP70 signal was thought to impair CAR-T cells functions (Gudipati et al., 2020). The ERK signal is considered as one of the main hubs to transduce proximal signals into nucleus during T cell activation. It is thought to exhibit a switch-like functions and the transient or persistent ERK dynamics could induce distinct gene expressions. The dynamics of ERK signal have been finely tuned by spatiotemporal networks to control cell fate decisions (Purvis and Lahav, 2013; Rohrs et al., 2020). Inside T cells, the ERK pathway is initiated by Rac-1 after the activation of Linker for activation of T cells (LAT), whose phosphorylation by ZAP70 makes it recruit multiple effector molecules for signal propagation. We found that TSHR binding elicits activation of ZAP70 and ERK in both CD28 and 4-1BB-based TSHR CAR-T cells. While two types of CAR-T cells had similar ZAP70 activity, different co-stimulatory signals resulted in distinctive ERK activation dynamics. This may be responsible for their different anti-tumor kinetics in mice.

## Materials and Methods

### Cell Lines

293T cells, Jurkat T cells and K562 cells were obtained from ATCC. Thyroid cancer cell line 8505c cells was obtained from DSMZ (German Collection of Microorganisms and Cell Cultures). 293T cells and 8505c cells were cultured in high glucose DMEM medium with 10% fetal bovine serum (FBS) and 1% penicillin/streptomycin (P/S). Jurkat T cells (clone E6-1) and K562 cells were cultured in IMDM medium with 10% FBS and 1% P/S. All cells were maintained in 5% CO_2_ at 37°C.

### Plasmids Construction

All plasmids were constructed using ClonExpress II One Step Cloning Kit (Vazyme, Nanjing, China) following manufacturer’s instruction. Retrovirus plasmids encoding TSHR-specific CARs were constructed by inserting the CAR constructs into SFG γ-retroviral vector. For detection of CAR expression, a sequence encoding green fluorescence protein (GFP) was linked in the C-terminus of CAR by a P2A peptide. Plasmids used for cell line transduction were constructed in lentiviral vectors. To prepare the plasmid co-expressing TSHR and luciferase, the sequences encoding TSHR and luciferase reporter were linked by a P2A peptide. The ZAP70 and ERK biosensors were reported by previous studies. The ZAP70 biosensor used the peptide (SREYACISGEL) as ZAP70 substrate (Liu et al., 2021). The ERK biosensor used the peptide (PDVPRTPVDKAKLSFQFPF) as ERK substrate (Komatsu et al., 2011). For co-expression of ZAP70 biosensor or ERK biosensor and CARs in Jurkat T cells, the CAR construct was introduced in the C-terminus of biosensor sequence using a P2A peptide.

### T Cell Isolation and Transduction

Human blood was obtained from healthy donors with written approval. PBMC was isolated using human lymphocyte isolation kit (Dakewe, Shenzhen, China). T cells were further purified using Pan T cell isolation kit (Miltenyi Biotec, Bergisch Gladbach, Germany) and cultured with X-VIVO 15 medium (Lonza) supplemented with 10% FBS, 1% P/S, 5 ng/ml IL-7 (Novoprotein, Shanghai, China) and 5 ng/ml IL-15 (Novoprotein). Immediately after T cell isolation, they were stimulated with CD3/CD28 T cell Activator Dynabeads (Invitrogen, Carlsbad, United States) at a ratio of 1:1. T cells transduction was performed after 48 h. Retrovirus was produced from 293T cell lines. T cells were transduced with retrovirus supernatants in retronectin (Takara, Otsu, Japan)-coated plates. To enhance transduction efficiency, the plates were centrifuged with 3000 rpm for 90 min.

### *In vitro* Cytotoxic Assay

CAR-T cells were co-cultured with 5×10^4^ target cells at different effector/target (E/T) ratio in target cell medium in black-walled 96 well plates. After 18 h, the cells were treated with D-luciferin (GoldBio, St. Louis, United States). Emitted light was detected by a luminescence plate reader (Thermo Varioskan Flash). Lysis percentage was calculated as [1-(luminescence intensity of each sample)/(luminescence intensity of target cells alone)] ×100.

### Flow Cytometry

For cytokines and exhaustion markers analysis, CAR-T cells were stimulated with irradiated 8505c-TSHR cells for 24 h. The BD Cytometric Bead Array (CBA) kit were used to quantify the secretion level of IL-2, TNFα and IFN-γ of the supernatant of CAR-T cells following manufacturer’s instruction. For intracellular cytokines staining, the Golgi plug protein transport inhibitor Brefeldin A (eBioscience, San Diego, United States) was added into the cultured medium 4 h before detection. T cells were then fixed and permeabilized with Fixation and Permeabilization kit (BD Biosciences, San Jose, United States) according to manufacturer’s instruction. The following antibodies were used: FITC mouse anti-human TSHR (Santa Cruz Biotechnology, Santa Cruz, United States), Human IL-2 Flex Set (BD Biosciences), Human TNF Flex Set (BD Biosciences), Human IFN-γ Flex Set (BD Biosciences), BV421 rat anti-human IL-2 (BD Biosciences), PE-Cy7 mouse anti-human TNFα (BD Biosciences), PE-Cy7 anti-human CD279 (PD-1) (eBioscience), eFluor 450 anti-human CD223 (LAG-3) (eBioscience). Flow cytometry was performed on a CytoFLEX LX cytometer (Beckman Coulter, Brea, United States). A moflo Astrios EQ cell sorter (Beckman Coulter) was used for cell sorting. Data were analyzed with FlowJo software (FlowJo LLC, Ashland, United States).

### Animal Experiments

Six- to 12-week-old NOD/SCID/IL-2Rγ^null^ (NSG) mice were obtained from Jihui Shanghai and housed in the Animal Core Facility at Westlake University. All procedures followed the Institutional Animal Care and Use Committee (IACUC) guideline. For the *in vivo* anti-tumor assay, mice were intravenously injected with 1×10^6^ 8505c-TSHR cells followed by 0.5×10^6^ CAR-T cells the next day. To monitor tumor burden, mice were treated with 200 μL of 15 mg/ml D-luciferin by intraperitoneal injection. Bioluminescence imaging was performed using the Optima small animal imaging system (Biospace Lab, Paris, France).

### Image Acquisition and Analysis

Time-lapse imaging was performed with a Nikon Eclipse Ti inverted microscope. A Tokai Hit ST Series Stage Top Incubator was used to maintain a 5% CO_2_ at 37°C for cells during imaging. The W-VIEW GEMINI imaging splitting optics (Hamamatsu Photonics, Hamamatsu, Japan) with a 438/29 nm excitation filter, a 474/40 nm emission filter, a 535/25 nm emission filter, an iXon Ultra 897 EMCCD camera (Andor Technology, Belfast, United Kingdom) was used to acquire the ECFP and FRET fluorescent signals simultaneously. CAR-Jurkat T cells co-expressing ZAP70 or ERK biosensor were generated by lentivirus transduction. To monitor ZAP70 or ERK signal dynamics upon antigen stimulation, CAR-Jurkat T cells were dropped on the glass-bottom dishes coated with K562-TSHR cells. From that time on, images were acquired at an interval of 30 s for 40 min. The imaging data were analyzed by Fluocell software. The ECFP/FRET ratio of ZAP70 biosensor or the FRET/ECFP ratio of ERK biosensor for each cell was normalized before comparison.

### Statistics

Statistical analysis was performed using GraphPad Prism 8 software. An unpaired, two-tailed student’s *t*-test were used to determine the statistical differences between two groups. A Kaplan-Meier curve and the log-rank test were used to compare the survival differences between the groups in animal experiments. A *p* value < 0.05 was considered to be statically significant.

## Results

### Design of CAR Constructs Targeting TSHR

**FIGURE 1:**
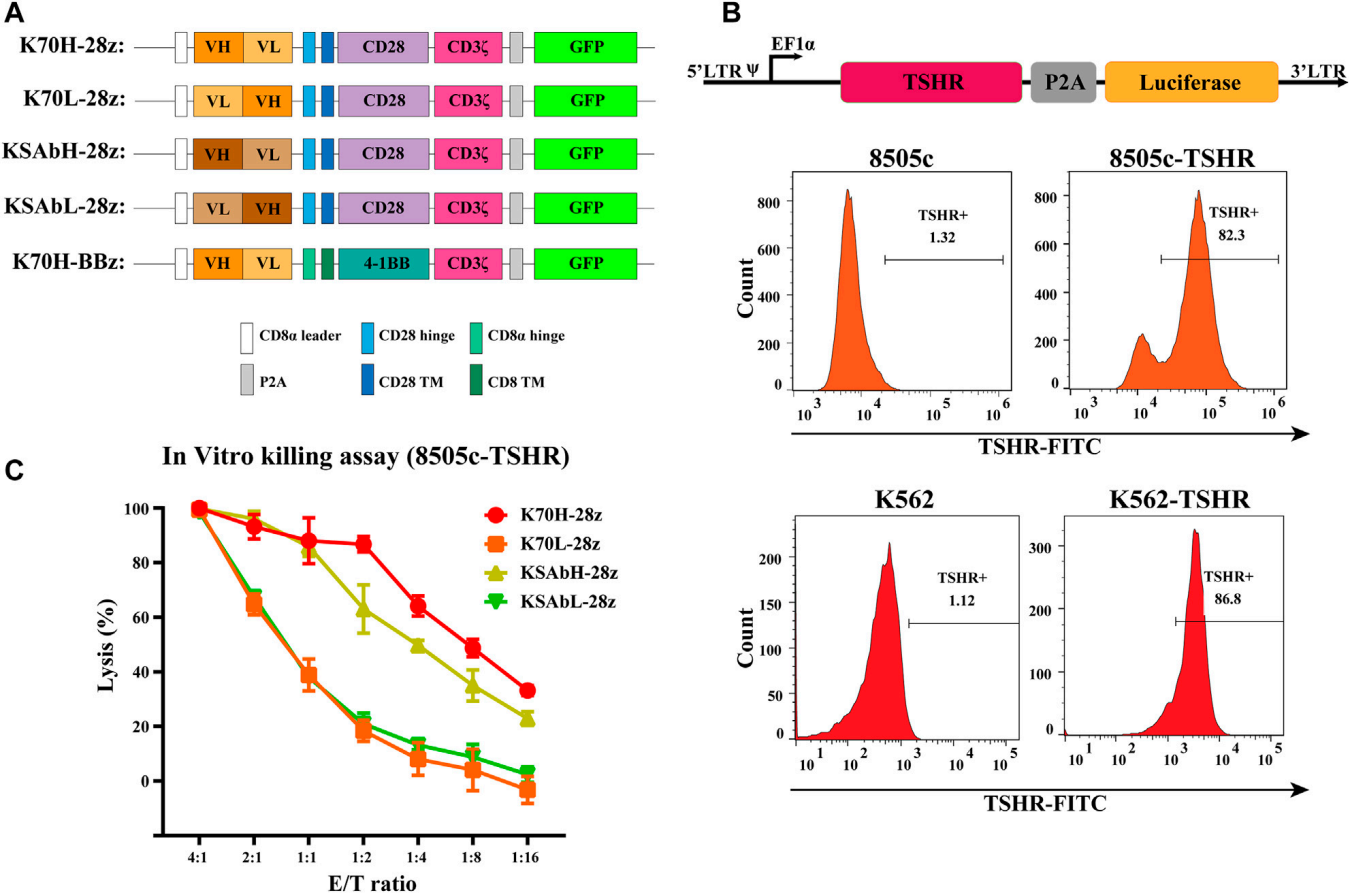
Design of TSHR-specific CARs. **(A)** Construction of CARs with different scFvs and co-stimulatory domains. TM: transmembrane. **(B)** Generation of 8505c and K562 cell lines expressing TSHR antigen. **(C)** *In vitro* killing assay of primary CAR-T cells with different scFvs. Error bars: mean ± SD.

Based on a second-generation CAR construct we investigated the feasibility of CAR-T cells in treating TSHR-positive thyroid cancers. In our design, the ectodomain of CARs was a single chain variable fragment (scFv) that recognize TSHR, and the endodomain contained a CD28 or 4-1BB-derived costimulatory domains and CD3ζ-derived activation domain. To evaluate the expression level of CARs, a green fluorescence protein (GFP) was introduced downstream of CAR through a P2A self-cleavage peptide (Figure 1A). Although not strictly correlated, the expression of GFP could be used to estimate CAR expression level. To find the optimized scFvs, we constructed and compared scFvs from two anti-TSHR monoclonal antibodies (K1-70 and KSAb) that have been previously reported. As the arrangement of variable heavy chain (VH) and variable light chain (VL) fragment may largely affect the antigen recognition property, scFvs with different VH-VL arrangements were constructed for each antibody. Thus, four CD28-based CAR constructs with different scFvs (referred to K70H-28z, K70L-28z, KSAbH-28z and KSAbL-28z) were generated (Figure 1A).

Next, we produced CD28ζ CAR-T cells with different scFvs from primary T cells and compared their cytotoxic effect. Since most thyroid cell lines have lost their TSHR expression (Li et al., 2021), we established a TSHR-positive target cell line by transducing the full-length TSHR gene with a luciferase reporter into the thyroid cancer cell line 8505c (8505c-TSHR) (Figure 1B). Using 8505c-TSHR as the target cell, *in vitro* killing assay revealed the superior cytotoxic effect of K70H-28z CAR-T cells than its counterparts (Figure 1C). Therefore, the K1-70 derived VH-VL scFv was selected for subsequent studies.

### *In vitro* Cytotoxicity of TSHR CAR-T Cells With CD28 or 4-1BB Co-Stimulatory Domain

**FIGURE 2:**
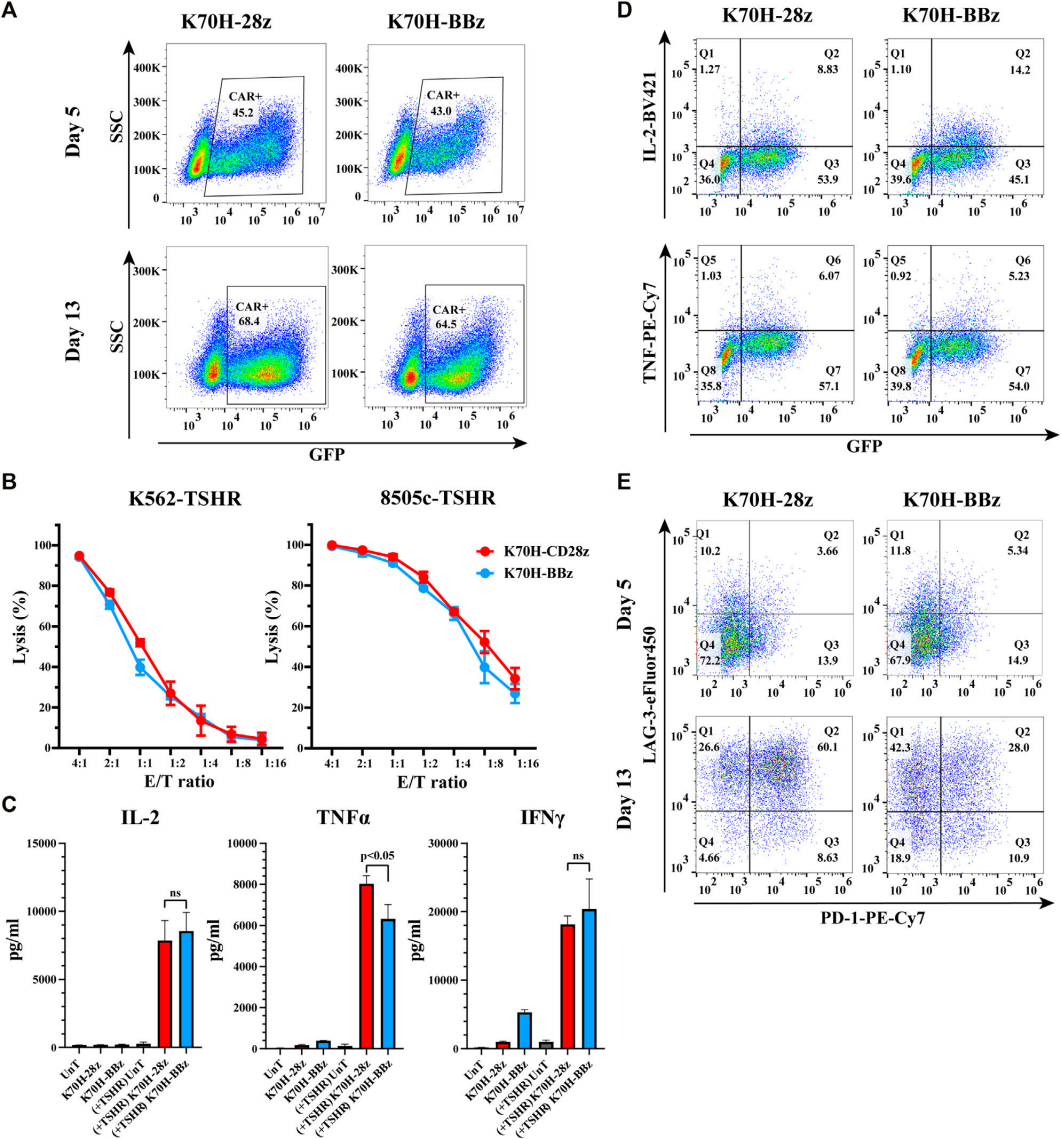
*In vitro* characterization of CD28 and 4-1BB-based TSHR CAR-T cells. **(A)** CAR expression of primary T cells after retrovirus transduction. T cells were collected at day 5 and 13 after PBMC isolation and evaluated by FACS. CAR expression was indicated by GFP intensity of T cells. **(B)** *In vitro* killing assay of different CAR-T cells against indicated target cells. **(C–E)** CAR-T cells were co-cultured with irradiated 8505c-TSHR cells for 24 h. The cytokine secretion of the supernatant **(C)**, the intracellular cytokines expression **(D)** and the exhaustion genes expression **(E)** of CAR-T cells were evaluated by FACS. UnT: Untransduced T cells. Error bars: mean ± SD.

Nowadays, the most widely used CARs in clinic contain either CD28 or 4-1BB as co-stimulatory domains. Both of them have achieved impressive results in clinical studies. However, CAR-T cells bearing CD28 or 4-1BB intracellular domains could have different cytotoxic functions and *in vivo* persistence (Feucht and Sadelain, 2020). To find out the optimal co-stimulatory domain for thyroid cancer treatment, we compared the *in vitro* anti-tumor functions of CD28 and 4-1BB-based TSHR CAR-T cells. The CAR structures (K70H-28z & K70H-BBz) have the same scFv but different hinge and transmembrane domains (Figure 1A). Produced by retrovirus transduction, both CAR-T cells showed similar transduction efficiency (Figure 2A). Using either K562-TSHR (Figure 1B) or 8505c-TSHR as target cells, their cytotoxicity was compared by *in vitro* killing assay. We found comparable cytotoxic effect between K70H-28z and K70H-BBz CAR-T cells (Figure 2B). We then analyzed the cytokines release of CAR-T cells by FACS. Co-culture with 8505c-TSHR cells promoted K70H-28z and K70H-BBz CAR-T cells but not the untransduced T cells to secrete robust IL-2, TNFα and IFNγ (Figure 2C). The K70H-28z CAR-T cells secreted comparable levels of IL-2 and IFNγ, but higher levels of TNFα than K70H-BBz CAR-T cells (Figure 2C). Intracellular cytokines staining further confirmed that antigen stimulation notably induced GFP^+^ CAR-T cells to secrete IL-2 and TNFα but not GFP^−^ T cells (Figure 2D).

After a period of *ex vivo* culture, which is often required for CAR-T cell expansion before infusion, we observed an increased GFP^+^ ratio of the total populations, representing an enrichment of the CAR-T cells. To evaluate the exhaustion levels, we detected PD-1 and LAG-3 expression of CAR-T cells after antigen stimulation (Figure 2E). Compared to CAR-T cells at day 5, CAR-T cells at day 13 showed increased expression of both exhaustion markers (Figure 2E). Moreover, 60.1% of the K70H-28z CAR-T cells showed double positive of PD-1 and LAG-3, much higher than that of the K70H-BBz group (Figure 2E).

### Similar ZAP70 Activation Downstream of CD28 or 4-1BB-Based TSHR CARs Revealed by FRET Imaging

**FIGURE 3:**
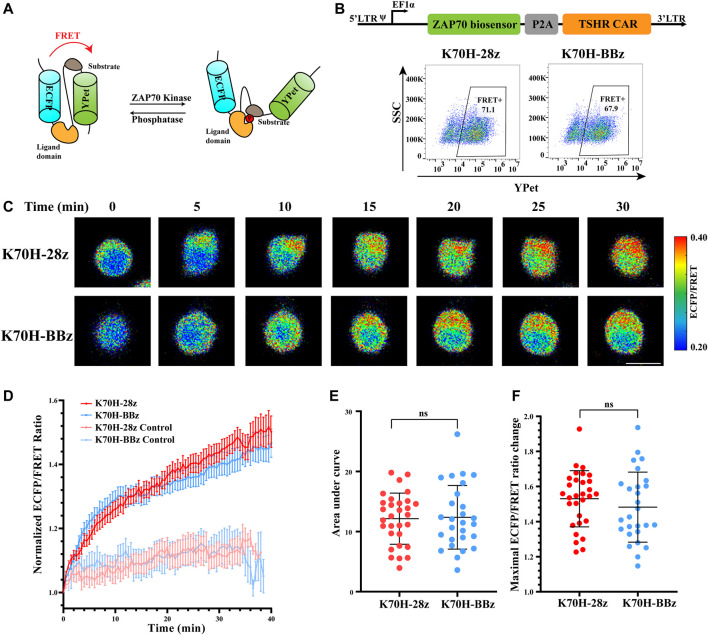
Signaling dynamics of ZAP70 in CAR-T cells upon antigen stimulation. **(A)** Design of ZAP70 FRET biosensor. **(B)** Biosensor (CAR) expression of Jurkat T cells transduced with ZAP70 biosensor and TSHR CARs. **(C)** ZAP70 signal changes of CAR-Jurkat T cells over time after contacting with K562-TSHR cells. The activity of ZAP70 was visualized by ECFP/FRET ratio. Scale bar: 10 μm. **(D)** Time courses of ECFP/FRET ratio of ZAP70 biosensor in K70H-28z or K70H-BBz CAR-Jurkat T cells stimulated by K562-TSHR (*n* = 30 and 27 respectively) or K562 (Control, *n* = 10 and 10 respectively). Error bars: mean ± SEM. Accumulated signal intensity (area under curve) **(E)** and maximal ECFP/FRET ratio changes **(F)** of ZAP70 biosensor in CAR-Jurkat T cells. Error bars: mean ± SD.

Although previous studies have characterized different signal activities triggered by CD28 or 4-1BB-based CARs (Philipson et al., 2020), their signaling dynamics in the early stage of T cell activation are still elusive. To figure out how co-stimulatory molecules affect CAR-induced signal transductions, we first focused on the membrane-proximal signaling events that were activated at the earliest time after antigen stimulation. ZAP70 has been considered as one of the most important kinases to transduce CAR signals into downstream signaling cascades (Wang et al., 2010; Gudipati et al., 2020). We have applied FRET-based ZAP70 biosensor to monitor the activity changes of ZAP70 during TSHR CAR-T cells activation. The ZAP70 biosensor was designed to include a FRET pair of ECFP and YPet fluorescence proteins, a ZAP70 kinase-specific substrate domain, and a ligand domain. Phosphorylation of the substrate by ZAP70 would cause conformational changes of the biosensor, resulting in reduction of FRET efficiency between ECFP and YPet (Figure 3A) (Liu et al., 2021). Therefore, the relative activity of ZAP70 could be evaluated by detecting the fluorescence ratio of ECFP and FRET (YPet). To assess the ZAP70 activity upon antigen stimulation, the Jurkat T cell line co-expressing ZAP70 biosensor and CARs (CAR-Jurkat T) were generated by lentivirus transduction. Both K70H-28z and K70H-BBz CAR-Jurkat T cells showed similar expression of biosensor (CAR) (Figure 3B). By FRET imaging, we observed remarkable changes of the ZAP70 activity in CAR-Jurkat T cells upon encountering with the K562-TSHR target cells (Figure 3C). The ZAP70 activity increased rapidly within 10 min after antigen stimulation. Thereafter, its signal continued to increase with slower speed (Figure 3D). We further quantified the accumulated signal intensity (area under curve) and maximal signal intensity (maximal ECFP/FRET ratio changes) of K70H-28z and K70H-BBz CARs. Neither the accumulated signal intensity nor the maximal signal intensity of ZAP70 between K70H-28z and K70H-BBz CARs showed significant difference (Figures 3E,F).

### Different ERK Activation Dynamics in TSHR CAR-T Cells Revealed by FRET Imaging

**FIGURE 4:**
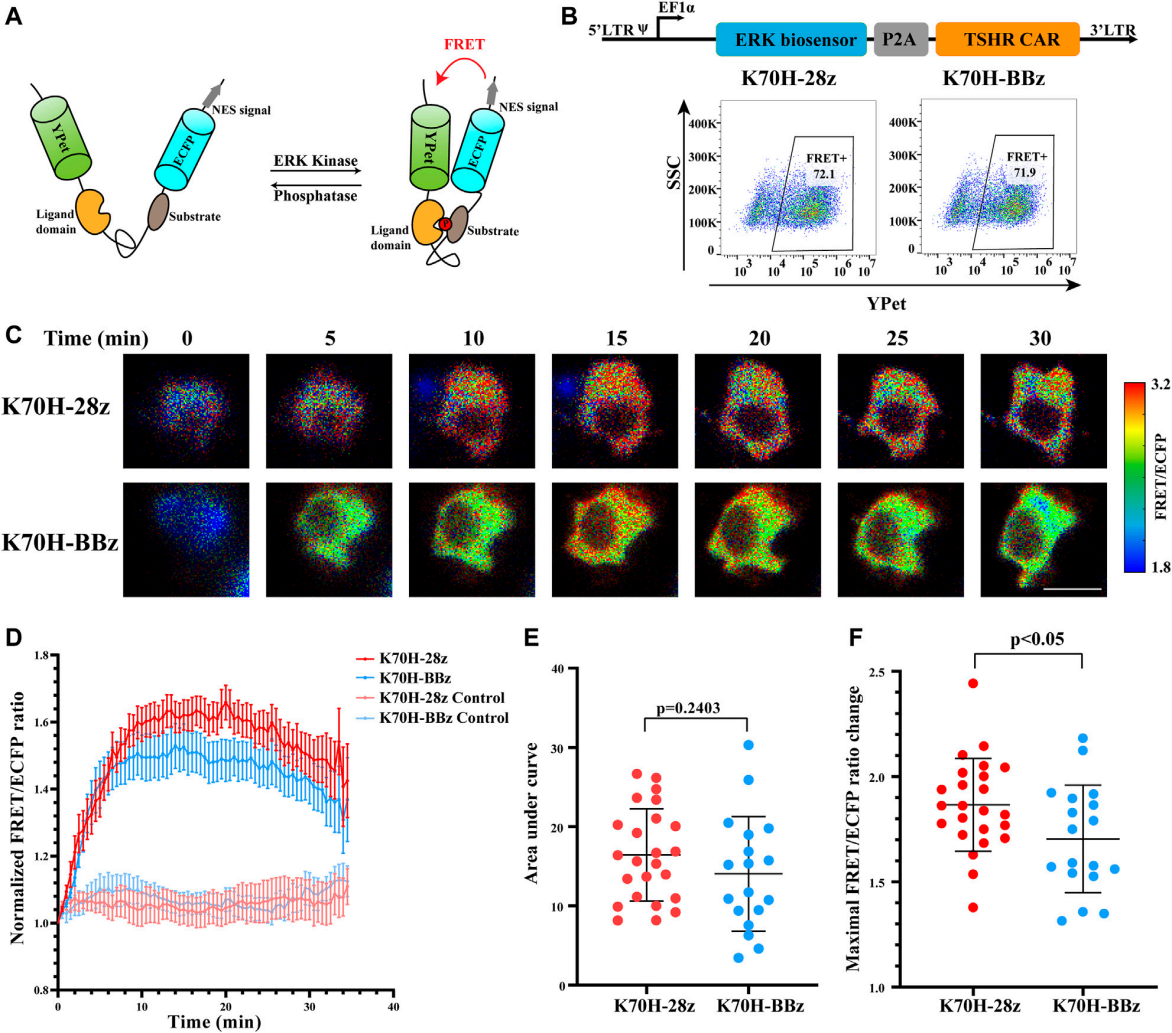
Signaling dynamics of ERK in CAR-T cells upon antigen stimulation. **(A)** Design of ERK FRET biosensor. **(B)** Biosensor (CAR) expression of Jurkat T cells transduced with ERK biosensor and TSHR CARs. **(C)** ERK signal changes of CAR-Jurkat T cells over time after contacting with K562-TSHR cells. The activity of ERK was visualized by FRET/ECFP ratio. Scale bar: 10 μm. **(D)** Time courses of FRET/ECFP ratio of ERK biosensor in K70H-28z or K70H-BBz CAR-Jurkat T cells stimulated by K562-TSHR (*n* = 24 and 18 respectively) or K562 (Control, *n* = 14 and 14 respectively). Error bars: mean ± SEM. Accumulated signal intensity (area under curve) **(E)** and maximal FRET/ECFP ratio changes **(F)** of ERK biosensor in CAR-Jurkat T cells. Error bars: mean ± SD.

To assess whether signals downstream of ZAP70 were affected by different co-stimulatory molecules, we investigated the signal dynamics of ERK during CAR-T cells activation by FRET-based biosensor. The ERK biosensor used a flexible linker to connect the substrate, ligand domain and FRET pairs. It also included a nucleus exporting signal (NES) to increase detection sensitivity of cytosolic signal. The biosensor would increase its FRET efficiency when phosphorylated by ERK (Figure 4A) (Komatsu et al., 2011). The CAR-Jurkat T cells expressing ERK biosensor and K70H-28z or K70H-BBz CAR were generated by lentivirus transduction and showed similar expression of biosensor (CAR) between groups (Figure 4B). By FRET imaging we found that the ERK activity, as visualized by FRET/ECFP fluorescence ratio of CAR-Jurkat T cells increased upon K562-TSHR cells stimulation (Figure 4C). Statistical quantification suggested that the activity of ERK reached to a peak level in about 15 min and then underwent slow decrease (Figure 4D). The accumulated signal intensity of ERK in K70H-28z CAR was slightly higher than that of K70H-BBz CAR but without significant difference (Figure 4E). However, the maximal signal intensity of ERK in K70H-28z CAR was significantly higher than that of K70H-BBz CAR (*p* < 0.05) (Figure 4F). Collectively, compared with K70H-BBz CAR, K70H-28z CAR triggered similar ZAP70 signaling but significantly enhanced ERK signaling.

### Different Anti-Tumor Kinetics of CD28 and 4-1BB-Based CAR-T Cells *in vivo*

**FIGURE 5:**
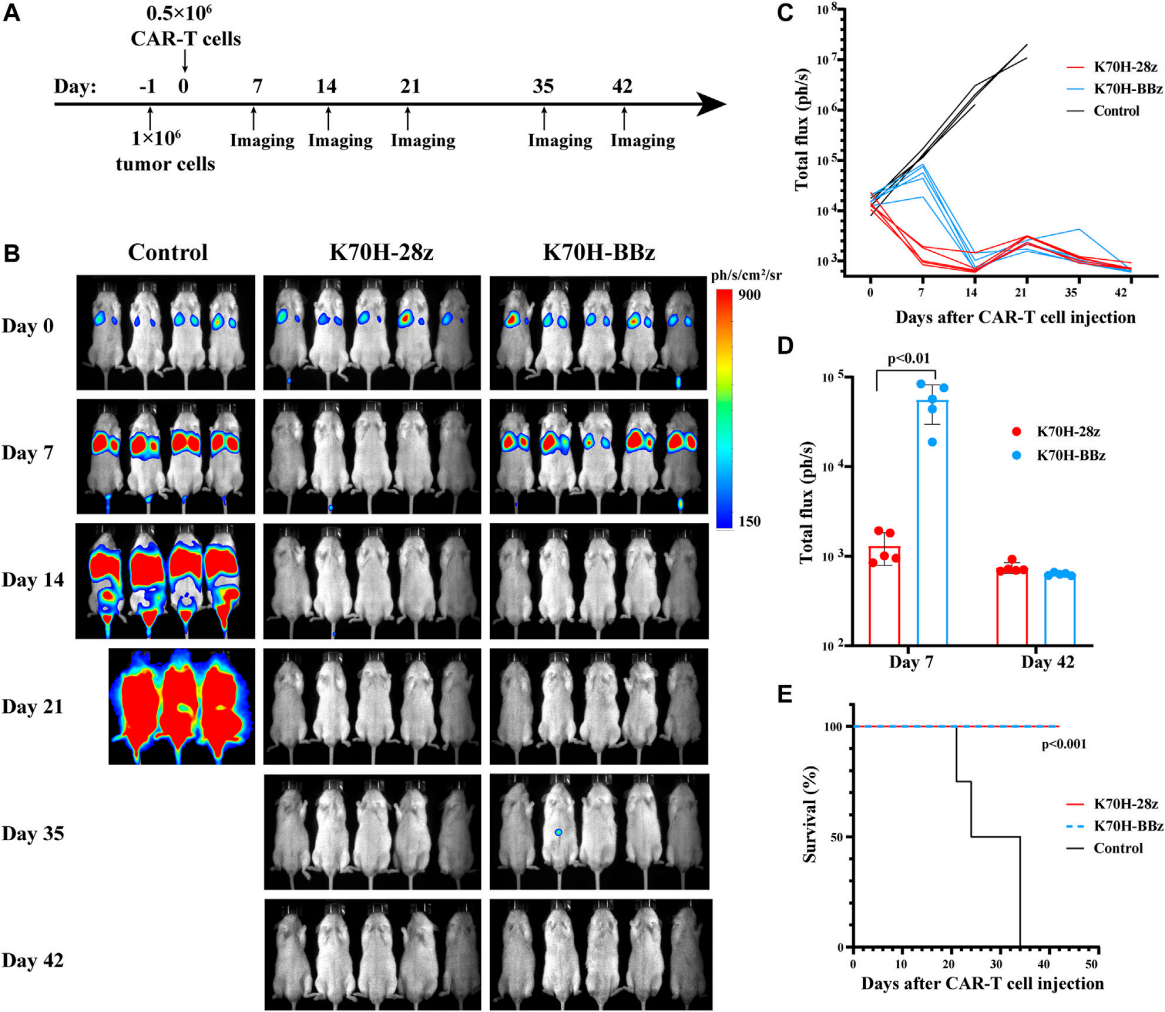
*In vivo* anti-tumor functions of TSHR CAR-T cells. **(A)** Schematic timeline of the *in vivo* experimental design. NSG mice were intravenously injected with 1×10^6^ 8505c-TSHR cells. The next day, they were treated with 0.5 × 10^6^ K70H-28z CAR-T cells (*n* = 5), K70H-BBz CAR-T cells (*n* = 5) or PBS (*n* = 4). **(B)** Bioluminescence imaging of mice at indicated days after treatment. **(C)** Quantification of tumor burden of mice. **(D)** Tumor burden of K70H-28z CAR and K70H-BBz CAR-T groups at day 7 and 42. Error bars: mean ± SD. **(E)** Kaplan-Meier analysis of survival of mice from different groups.

Finally, we evaluated the anti-tumor functions of K70H-28z and K70H-BBz CAR-T cells by *in vivo* studies (Figure 5A). To mimic the distant metastasis of DTCs, of which lung and bone are the major sites, we established a xenograft animal model based on previous studies (Zhang et al., 2014). The 8505c-TSHR cells were intravenously injected into the immunodeficient NSG mice. We found the tumors cells accumulated in the lung at early stage. Bone and other tissue metastasis happened after 14 days and caused animal death in the control group (Figures 5B,C,E). By contrast, mice treated with either K70H-28z or K70H-BBz CAR-T cells successfully controlled tumor growth and survived without obvious side-effect (Figures 5B,C,E). Nevertheless, K70H-28z CAR-T cells were found to eradicate nearly all tumor cells within 7 days, while K70H-BBz CAR-T cells lagged behind (Figures 5C,D). This indicated faster anti-tumor kinetics of K70H-28z CAR-T cells than K70H-BBz CAR-T cells.

## Discussion

In this study, we developed a CAR-T therapy for TSHR-positive advanced thyroid cancers. The CAR constructs were designed to bear a monoclonal antibody-derived scFv targeting TSHR and a CD28 or 4-1BB co-stimulatory domain. Primary T cells equipped with the CD28 or 4-1BB-based CARs were demonstrated with potent anti-tumor efficacy both *in vitro* and *in vivo*. Moreover, we applied the FRET biosensors for ZAP70 and ERK to monitor the signaling dynamics of CAR-T cells in response to antigen stimulation. These results revealed different ERK activation properties of CD28 and 4-1BB-based CAR-T cells.

To recognize TSHR-positive tumor cells, we constructed scFvs from two monoclonal antibodies, K1-70 and KSAb. Both antibodies were derived from patients with Graves’ disease. They have an estimated affinity of 25 and 22 pM for human TSHR, respectively (Sanders et al., 2010; Banga et al., 2013). The slightly higher affinity of K1-70 may contribute to the stronger cytotoxicity of K70H-28z CAR-T cells than KSAbH-28z CAR-T cells. Despite the potent anti-tumor effect of K70H-derived CAR-T cells, previous studies have suggested that the affinity of scFvs did not positively correlate with CAR-T cells functions (Ghorashian et al., 2019). The increased affinity of scFvs above threshold may not improve T cell activation but decrease selectivity. Fine-tuning of the scFv affinity may be required to maximize CAR-T cell functions as well as minimize possible on-target/off-tumor toxicity (Di Roberto et al., 2020).

To transduce the antigen stimulation into intracellular signals responsible for T cell activation, the cytoplasmic domains of CARs have been incorporated with a CD3ζ chain and a CD28 or 4-1BB derived co-stimulatory domain. Previous studies have suggested remarkable variations in functions of CAR-T cells equipped with various co-stimulatory domains. In CD19 CAR-T cells studies, 28z CAR-T cells exhibited higher effector functions with less persistence than BBz CAR-T cells (Shah and Fry, 2019; Ying et al., 2019). This functional discrepancy has been associated with different metabolic features. 28z CAR-T cells have shown enhanced aerobic glycolysis, while BBz CAR-T cells have exhibited greater mitochondrial oxidative phosphorylation (Kawalekar et al., 2016). Interestingly, phosphoproteomic analysis have indicated that 28z CAR and BBz CAR induce T cells activation through common signaling intermediates but with different kinetics and intensities (Salter et al., 2018).

We detected the ZAP70 and ERK signal activities in CAR-Jurkat T cells after antigen stimulation by FRET biosensors. Compared with methods like Western blot and mass spectrum, FRET biosensors could detect kinase activity in single live cells with high spatiotemporal resolution. This enables us to evaluate early signaling events of T cell activation. We observed the interaction with TSHR-positive tumor cells induced rapid increase of ZAP70 and ERK activities within minutes, indicating antigen-dependent activation of the CAR-Jurkat T cells. Different from previous studies, we did not observe significant difference of ZAP70 signal activities in K70H-28z and K70H-BBz CAR-T cells after activation (Salter et al., 2018; Philipson et al., 2020). One main reason responsible for these results may be the different detection methods. In previous studies, ZAP70 activity was evaluated by the phosphorylation level of some key phosphorylated sites like Y319. However, the phosphorylation state of the specific site did not precisely reflect the activity of kinase. In contrast, the FRET ratio change of FRET biosensors was determined by the phosphorylation of ZAP70-specific substrate, which could more accurately reflect the activation state of ZAP70. In addition, the previous comparison was done between CD19-targeting 28z and BBz CAR-T cells while in this study, TSHR-targeting CAR-T cells have different scFvs and CAR targets. Nevertheless, ZAP70 activation mainly depends on CD3ζ instead of co-stimulatory molecules, which matched our results.

Consistent with previous studies, we observed an enhanced signal activity of ERK in 28z CAR-T cells when compared to BBz CAR-T cells (Salter et al., 2018). CD28 and 4-1BB as co-stimulatory molecules are thought to amplify ERK signaling with distinct mechanisms. CD28 may enhance ERK signaling by promoting the recruitment of Vav-1 to the plasma membrane, which is required for Rac-1 activation (Michel et al., 2000). 4-1BB may induce ERK activation via its downstream receptor TRAF2 to regulate Tpl2 activation (Xie et al., 2021). In CAR-T cells it is still elusive how CARs with different co-stimulatory molecules mediate the activation of ERK signal. Diverse signaling output derived from CD28 or 4-1BB may contribute to the amplification of ERK pathway. As observed in our studies, the different ERK activities of K70H-28z and K70H-BBz CAR-T cells may induce large distinction of downstream signals and contribute to different anti-tumor functions. Notably, we have observed that the ERK signal reached a peak value at about 15 min after antigen engagement, at which time ZAP70 signal also showed a short plateau. Thereafter, the increased ZAP70 activities did not promote further enhancement of ERK signals. The correlation of ZAP70 and ERK signals requires further investigation. In addition, single cell FRET imaging allowed us to observe heterogeneous activities of ZAP70 and ERK in individual CAR-T cells after antigen stimulation. This cell-to-cell heterogeneity obscured by traditional methods may be caused by distinct expression levels of CARs and related signaling molecules or different contacting strength with target cells. More importantly, this heterogeneity may lead to the diverse T cell differentiation and fates. However, with limited number of cells collected by FRET imaging, it is challenging to find possible links and correlations.

Although with comparable cytotoxic effects *in vitro*, we have shown that K70H-28z CAR-T cells had higher expression of the exhaustion genes than K70H-BBz CAR-T cells. This may suggest the shorter persistence of K70H-28z CAR-T cells. Meanwhile, the increased PD-1 and LAG-3 expression could be an indicator of T cell activation and the result of rapid effector functions of K70H-28z CAR-T cells (Wherry and Kurachi, 2015). Nevertheless, our *in vivo* experiments have observed potent anti-tumor functions of both designed CAR-T cells. Moreover, compared to K70H-BBz CAR-T cells, K70H-28z CAR-T cells have shown rapid elimination of tumor cells, which suggested higher effector functions. It should be noticed that although long-term persistence of CAR-T cells *in vivo* is essential to avoid tumor relapse, it may also enhance the risk of off-tumor toxicity (Kawalekar et al., 2016). In the context of thyroid cancer treatment, it requires further studies to balance the effector functions and long-term persistence of CAR-T cells.

Finally, our studies have shown the efficacy of TSHR CAR-T cells in the treatment of TSHR-positive thyroid cancers. We also showed that CD28 and 4-1BB-based CAR-T cells exhibited comparable anti-tumor functions with different kinetics. Live cell imaging using FRET biosensors revealed the dynamics of ZAP70 and ERK activities in both CAR-T cells during activation. These results promoted our understanding of CAR-T cells signaling and indicated potential applications of FRET biosensors in optimizing CAR designs. CAR-T cells therapy may overcome current obstacles for thyroid cancer treatment in the near future.

## Data Availability

All data obtained and/or analyzed during the current study are available from the corresponding authors on reasonable request.
